# Toxicities Associated with Continuing Disease-Modifying Antirheumatic Drugs for Collagen Vascular Diseases During Head and Neck Intensity-Modulated Radiotherapy: A Single-Institution Experience

**DOI:** 10.1177/19160216251379333

**Published:** 2025-09-25

**Authors:** Lisa Ni, Christina Phuong, Sue S. Yom, Jason W. Chan

**Affiliations:** 1 Department of Radiation Oncology, Helen Diller Comprehensive Cancer Care Center, University of California, San Francisco, Seattle, WA, USA

**Keywords:** quality of life, connective tissue disorders, radiation therapy, head and neck, outcomes, collagen vascular diseases

## Abstract

**Importance:**

Limited evidence exists to guide the safe use of radiotherapy (RT) and concurrent disease-modifying antirheumatic drugs (DMARDs) in patients with collagen vascular disease (CVD).

**Objective:**

To describe toxicity outcomes in patients with CVD receiving intensity-modulated radiotherapy (IMRT) for head and neck cancer (HNC) and to evaluate whether concurrent DMARD use is associated with increased toxicity.

**Design:**

Retrospective cohort study.

**Setting:**

Single academic tertiary care center in the United States, from 2005 to 2022.

**Participants:**

Twenty-three adult patients with CVD [eg, rheumatoid arthritis (RA), systemic lupus erythematosus (SLE), dermatomyositis (DM)] and biopsy-proven HNC treated with curative-intent IMRT. Eligibility required available treatment records and ≥90 days of follow-up post-RT.

**Intervention or Exposures:**

Definitive or postoperative IMRT for HNC. Exposure of interest was concurrent use of DMARDs during RT.

**Main Outcome Measures:**

Rates of acute (≤90 days) and late (>90 days) grade ≥2 and ≥3 toxicities, as graded by CTCAE v5.0. Fisher exact tests were used to compare toxicity rates by DMARD use.

**Results:**

Median follow-up was 56 months (IQR 9-98). Most common CVDs were RA (39%), SLE (17%), and DM (17%). Median RT dose was 66 Gy (range 48-70 Gy); 39% received concurrent chemotherapy. Acute grade ≥3 toxicity occurred in 35% (n = 8) and late grade ≥3 in 13% (n = 3). No grade ≥4 toxicities were observed. DMARD use during RT was not associated with higher rates of acute or late grade ≥2 or ≥3 toxicity (*P* > .1 for all comparisons).

**Conclusions:**

IMRT was associated with moderate rates of severe toxicity in patients with CVD, but DMARD use during RT did not increase risk.

**Relevance:**

Concurrent DMARDs may be safely continued during IMRT for HNC in patients with CVD. Prospective studies are needed to confirm these findings and refine risk stratification by CVD subtype and treatment regimen.

## Graphical Abstract


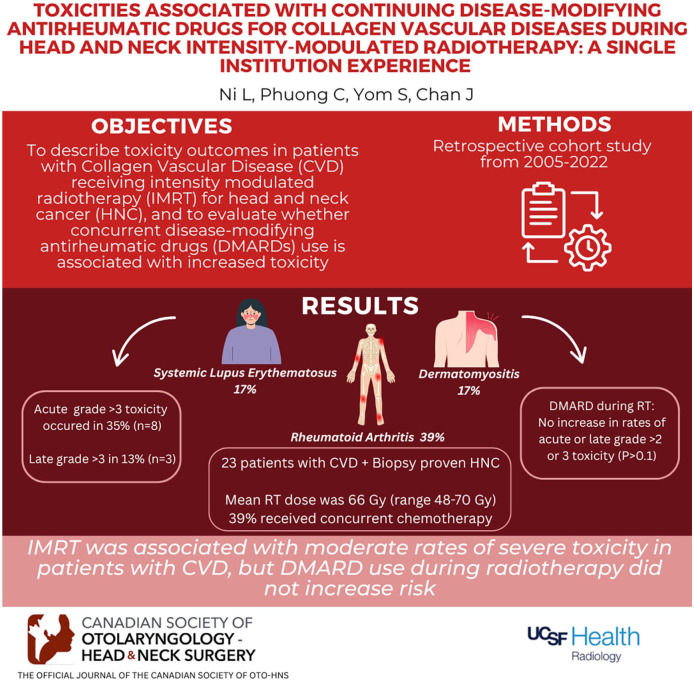


## Key Messages

1. Intensity-modulated radiotherapy (IMRT) is tolerable in patients with collagen vascular disease (CVD).
2. Use of conventional or biological disease-modifying antirheumatic drugs (DMARDs) during IMRT was not associated with increased acute or late toxicities, suggesting they can be continued safely during treatment.
3. Risk factors for acute toxicity include concurrent chemotherapy and higher biologically-effective dose (BED) but not DMARD use.

## Introduction

Collagen vascular diseases (CVDs), also known as connective tissue disorders, are a diverse group of immunologically-mediated systemic inflammatory disorders. Historically, CVDs were considered at least a relative contraindication to the use of radiotherapy (RT), due to reports of a disproportionately-high risk for severe toxicities such as fibrosis and necrosis in patients with CVDs.^1-3^ As such, radiation oncologists may still be hesitant to recommend definitive RT in patients with comorbid CVD and cancer diagnoses.

Recently-published meta-analyses suggest that the risk for severe toxicities in patients with CVDs receiving RT is not necessarily higher for all patients with CVDs when compared with patients without CVDs, but instead is likely related to multiple factors, including CVD subtype, treatment site, and radiation dose.^4-6^ For example, some studies indicate that for patients with certain CVDs such as rheumatoid arthritis (RA), there may not be an increased risk for severe toxicities, while for patients with CVDs such as systemic lupus erythematosus (SLE), dermatomyositis (DM), and scleroderma, there may be as high as a 2- to 4-fold risk for severe toxicities compared with patients without CVD.^2,7-9^ There are also data to suggest that patients with head and neck cancer and comorbid CVD are at especially high risk of acute and late toxicities from RT.^6,10,11^ More studies are needed to determine which CVDs are absolute contraindications to head and neck RT.

Furthermore, an important question that remains is whether to continue disease-modifying antirheumatic drugs (DMARDs) in patients with CVD receiving RT. DMARDs are immunomodulatory agents that may diminish the effect of anticancer therapies. Some DMARDs such as methotrexate impair DNA synthesis and may worsen side effects and delay recovery from RT. Currently, very few studies even report on the concurrent use of DMARDs during RT, and to our knowledge, no guidelines exist to inform the use of DMARDs in patients with CVD during RT.

The purpose of this study was to describe a single institution experience of treating patients with head and neck cancer (HNC) with definitive or postoperative intensity-modulated radiotherapy (IMRT) in the setting of comorbid CVD and to evaluate whether the use of concurrent DMARDs during HNC RT is safe in this patient population.

## Materials and Methods

Institutional review board approval was obtained for this retrospective study. Patients diagnosed with CVD who were treated with IMRT for cancer of the head and neck between January 2005 through December 2022 were included. Patients were identified by searching an institutional cancer registry electronic database for the diagnoses of CVDs confirmed by their rheumatologist including rheumatoid arthritis, systemic lupus erythematosus, systemic scleroderma, polymyositis, dermatomyositis, Sjögren’s syndrome, mixed connective tissue disease, psoriatic arthritis, and sarcoidosis. This cohort of patients was then cross-referenced with a radiation oncology departmental electronic database to identify patients treated with IMRT for cancer involving the head and neck at a single-institution tertiary academic center. Patients who received RT with palliative intent were excluded.

Data were manually abstracted from electronic medical records, including patient demographic, clinical, and treatment characteristics. CVD features abstracted included primary diagnosis, age at diagnosis, CVD symptom burden at the time of IMRT as defined by their rheumatologists, and the receipt of steroidal medications or DMARDs at the time of IMRT.

Acute and late toxicities were retrospectively classified using Common Terminology Criteria for Adverse Events (CTCAE) version 5.0, with acute toxicity defined as those reported within 90 days of completion of IMRT.^12^ Toxicity rates were compared across groups using Fisher exact tests. *P* values < .05 were considered statistically significant.

## Results

### Patient Clinical and Treatment Characteristics

Patient demographics and treatment characteristics are included in Table 1. Median follow-up from end of RT was 56 months (interquartile range: 9-98 months). Median age at the start of RT was 59 years (range: 36-87 years). Of the patients, 15 (65%) were female and 8 (35%) were male. Thirteen (57%) patients were white, and 10 (43%) patients were non-white. Eleven (48%) patients had a former smoking history (range: 2-38 pack-years), and 12 (52%) patients were never-smokers. Fourteen (61%) patients had mucosal squamous cell carcinoma (SCC), 3 (13%) had cutaneous SCC, 2 (9%) had nasal cavity/paranasal sinus tumors, 2 (9%) had salivary gland tumors, 1 (4%) had cutaneous melanoma, and 1 (4%) had mucosal melanoma.

**Table 1.** Patient and Treatment Characteristics.

Characteristic	N (%)
Age at the time of RT, years	
Median (range)	59 (36-87)
Age at CVD diagnosis, years	
Median (range)	50 (10-81)
Sex	
Female	15 (65)
Male	8 (35)
Race	
White	13 (57)
Non-white	10 (43)
Smoking status	
Never	12 (52)
Former	11 (48)
Disease site	
Nasopharynx	4 (17)
Oral Cavity	3 (13)
Oropharynx	4 (17)
Larynx	3 (13)
Nasal cavity / paranasal sinuses	2 (9)
Skin	4 (17)
Histology	
Mucosal squamous cell carcinoma	14 (61)
Cutaneous squamous cell carcinoma	3 (13)
Salivary gland tumors	2 (9)
Nasal cavity / paranasal sinus tumors	2 (9)
Mucosal melanoma	1 (4)
Cutaneous melanoma	1 (4)
Disease status	
Primary	20 (87)
Recurrent	3 (13)
Prior H&N RT	
No	22 (96)
Yes	1 (4)
CVD type	
Rheumatoid arthritis	9 (39)
Systemic lupus erythematosus	4 (17)
Dermatomyositis	4 (17)
Psoriatic arthritis	2 (9)
Polymyositis	2 (9)
Mixed connective tissue disorder	1 (4)
Other	2 (9)
CVD symptom burden at the time of RT	
None	17 (74)
Mild	5 (22)
Moderate to high	1 (4)
Taking DMARDs at the time of RT	
Yes	15 (65)
No	8 (35)
DMARD type at the time of RT	
Methotrexate	4 (17)
Mycophenolate mofetil	4 (17)
Hydroxychloroquine	4 (17)
Sulfasalazine	2 (9)
Other conventional DMARD	2 (9)
Biological DMARD	4 (17)
Taking multiple CVD meds at the time of RT	
Yes	10 (43)
No	13 (57)
RT Setting	
Adjuvant	14 (61)
Primary	9 (39)
Elective nodal RT	
Yes	18 (78)
No	5 (22)
PEG tube	
Yes	6 (26%)
No	17 (74%)
BED-3, Gy	
Median (range)	114 (86-119)
BED-10, Gy	
Median (range)	81 (59-85)
Planning target volume, cc	
Median (range)	65 (13-345)
Concurrent chemotherapy	
Yes	9 (39)
No	14 (61)

Abbreviations: “RT”, radiotherapy; “CVD”, collagen vascular disease; “H&N”, head and neck; “DMARD”, disease-modifying antirheumatic drug; “BED”, biologically-effective dose; “BED-3”, BED with *α / β* ratio of 3; “BED-10”, BED with *α / β* ratio of 10.

Total N = 23 patients.

The most common CVD diagnoses were RA (9 patients, 39%), SLE (4 patients, 17%), and DM (4 patients, 17%). Median age at the diagnosis of CVD was 50 years (range: 10-81 years). At the time of RT, 17 (74%) patients had no CVD symptoms, while 5 (22%) patients had a mild CVD symptom burden, and 1 (4%) patient had a moderate-to-high CVD symptom burden. At the time of RT, 15 (65%) patients were on DMARDs, and 11 (48%) patients were on multiple concurrent medications for the management of their CVD, including corticosteroids or conventional or biological DMARDs. The most common conventional DMARDs at the time of RT were methotrexate (4 patients), mycophenolate mofetil (4 patients), and hydroxychloroquine (4 patients). Four patients (17%) were taking biological DMARDs at the time of RT.

In terms of RT indication, 9 (39%) patients were treated with definitive RT and 14 (61%) were treated with postoperative RT. Twenty (87%) patients were treated at the first presentation of their HNC, while 3 (13%) were treated in the recurrent setting, and of these patients, 1 (4%) patient had received prior RT to the head and neck region. Concurrent chemotherapy was administered to 9 (39%) patients. Median total IMRT prescription dose was 66 Gy (range: 48-70 Gy), in 1.8 to 2.4 Gy fractions. Median total BED (Gy_3_) was 114.4 Gy_3_ (range: 86.4-119.4 Gy_3_), and median total BED (Gy_10_) was 80.5 Gy_10_ (range: 59.5-84.8 Gy_10_).

### Acute Toxicities

Fifteen (65%) patients experienced acute grade 2 or higher toxicities. Twelve (52%) patients experienced acute grade 2 toxicities and 8 (35%) patients experienced acute grade 3 toxicities. Acute grade 2 toxicities included mucositis (5 patients), dysphagia (5 patients), dry mouth (3 patients), dysgeusia (3 patients), nausea (2 patients), and radiation dermatitis (4 patients). Acute grade 3 toxicities included mucositis (4 patients) and nausea (4 patients). No patients experienced acute grade 4 or higher toxicities. Percutaneous endoscopic gastrostomy (PEG) tubes were placed in six patients (26%), including one placed prophylactically. Clinical and treatment details for patients who experienced acute and late grade 3 toxicities are included in Table 2.

**Table 2.** Characteristics of Patients Who Had Acute or Late Grade 3 Treatment-Related Toxicity.

Age (Years)	Sex	Primary Site	CVD Type	RT Setting	Dose (Gy)/ No. of Fx	Concurrent Chemo	On DMARD At Time Of RT	Elective Nodal RT	Acute Grade 3 Toxicity	Late Grade 3 Toxicity
75	F	Nasopharynx	DM	Primary	70/35	No	No	Yes	Mucositis	-
52	M	Nasopharynx	RA	Primary	69.96/33	Yes	Yes	Yes	Mucositis	-
36	F	Nasopharynx	Polymyositis	Primary	69.96/33	Yes	Yes	Yes	Mucositis; Dermatitis	-
45	F	Oropharynx	Sarcoidosis	Primary	69.96/33	Yes	No	Yes	Nausea	-
66	F	Oropharynx	RA	Adjuvant	66/33	Yes	Yes	Yes	Mucositis	-
55	F	Paranasal Sinus	RA	Adjuvant	59.4/33	No	Yes	No	Nausea	-
52	F	Parotid	SLE	Adjuvant	66/30	No	No	Yes	Nausea	-
73	F	Nasal Cavity	RA	Adjuvant	60/30	Yes	Yes	No	Nausea	-
56	M	Larynx	SLE	Primary	70/35	Yes	Yes	Yes	-	Laryngeal Edema
62	M	Oropharynx	DM	Adjuvant	69.96/33	Yes	No	Yes	-	Trismus
58	F	Oral Cavity	RA	Adjuvant	60/30	No	Yes	No	-	Osteonecrosis Of Jaw

Abbreviations: F, female; M, male; DM, dermatomyositis; RA, rheumatoid arthritis; SLE, systemic lupus erythematosus.

### Late Toxicities

**Figure 1.**
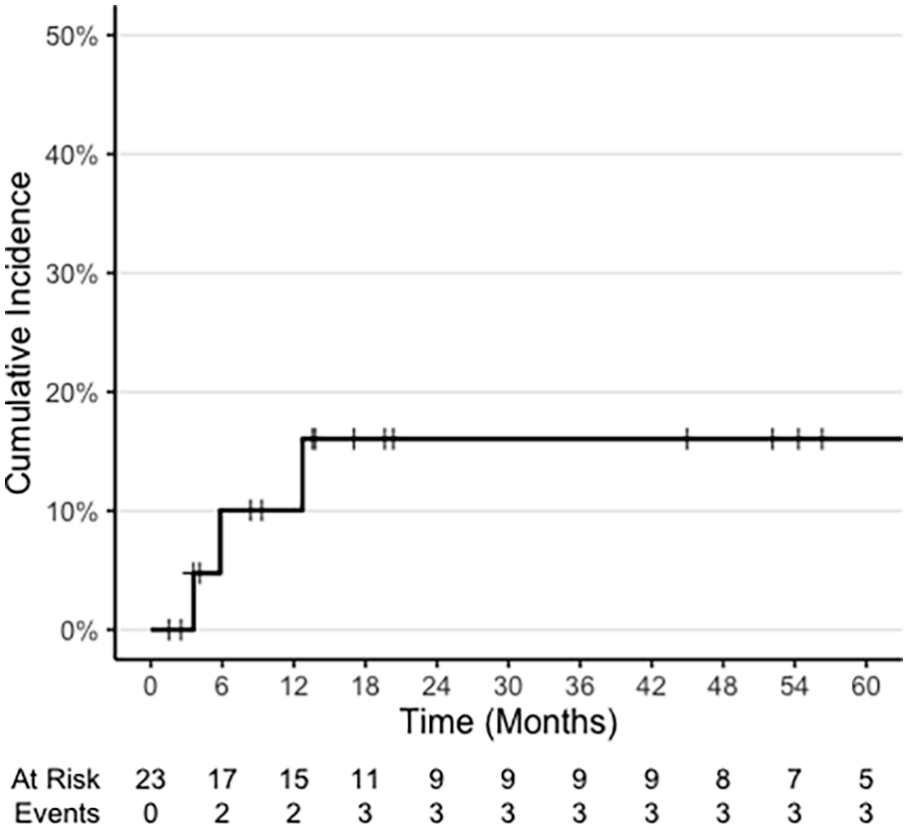
Cumulative incidence of late grade 3 toxicities in patients with head and neck cancers with comorbid collagen vascular disease treated with intensity-modulated radiotherapy. N = 23 patients.

Five (22%) patients experienced late grade 2 or higher toxicities. Three (13%) patients experienced late grade 2 toxicities, and 3 (13%) patients experienced late grade 3 toxicities (Figure 1). Of the 5 patients who experienced late grade 2 or high toxicities, 4 (80%) patients were taking DMARDs, and 4 (80%) patients were taking multiple medications for the treatment of their CVD at the time of HNC RT.

In terms of late grade 2 toxicities, Patient 5, who had RA with low symptom burden at the time of RT and underwent primary chemoradiation for nasopharyngeal carcinoma, experienced late grade 2 dysgeusia affecting their diet, which was present after the completion of treatment and was slowly improving up to their last follow-up 20 months after RT. Patient 8, who had RA that was controlled at the time of RT and underwent primary RT alone for supraglottic SCC, experienced late grade 2 laryngeal edema causing throat pain, which developed 6 months after RT and was intermittently present until last follow-up 17 months after RT.

In terms of late grade 3 toxicities, Patient 3, who had DM (at baseline associated with swallowing difficulty) with no CVD symptom burden at the time of RT, underwent primary chemoradiation for oropharyngeal SCC after an aborted transoral robotic surgery (TORS), and experienced late grade 2 dysarthria, which was present after the completion of treatment and was still present at last follow-up 5 years after RT. This patient also developed late grade 3 trismus starting 13 months after RT, which compounded their difficulty getting adequate nutrition in the setting of their DM, eventually requiring gastrostomy tube placement 3 years after RT. Patient 2, who had SLE with a moderate-to-high symptom burden at the time of RT, underwent primary chemoradiation for glottic SCC and experienced late grade 3 laryngeal edema 6 months after RT contributing to worsening aspiration and difficulty breathing, eventually undergoing a planned tracheostomy 10 months after RT. Patient 20, who had RA that was controlled at the time of RT, underwent anterior floor of mouth excision and bilateral neck dissection followed by adjuvant RT for mucosal melanoma involving the floor of mouth, and experienced grade 3 osteonecrosis of the jaw, which developed 4 months after RT, eventually requiring mandibular resection and free flap reconstruction 18 months after RT.

### Univariate Analysis for Acute and Late Toxicities

**Figure 2.**
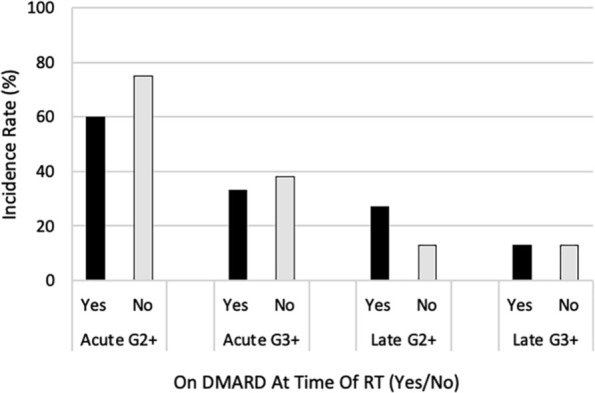
Incidence rates of acute and late toxicity, grouped by whether patients were taking disease-modifying antirheumatic drug (DMARD) at the time of radiotherapy (RT). Abbreviations: G2+, grade ≥ 2 toxicity; G3+, grade ≥ 3 toxicity.

Results of the univariate analysis are shown in Table 3. A higher BED (≥ 80.5 Gy_10_) was significantly associated with a higher rate of acute grade ≥ 2 toxicities (92% vs 30%, *P* = .006). Receipt of concurrent chemotherapy with RT was also significantly associated with a higher rate of acute grade ≥ 2 toxicities (100% vs 43%, *P* = .007). Factors associated with numerically, but not statistically, significant higher rate of acute grade ≥ 2 toxicities were are as follows: primary vs. adjuvant RT (89% vs 50%, *P* = .09), low/moderate/high CVD symptom burden vs. no CVD symptom burden at the time of RT (100% vs 53%, *P* = .06), and not being on multiple medications for CVD management at the time of RT (50% vs 77%, *P* = .22). No variables were associated with a statistically-significant increase in the rate of acute grade 3 toxicities. Factors associated with numerically, but not statistically, significant higher rate of acute grade 3 toxicities were as follows: female vs. male sex (47% vs 13%, *P* = .18) and concurrent chemotherapy (56% vs 21%, *P* = .18). The use of DMARDs (yes vs. no) during RT was not associated with a higher rate of acute grade ≥ 2 toxicities (60% vs 75%, *P* = .66) or acute grade ≥ 3 toxicities (33% vs 38%, *P* = 1.00; Figure 2). Among patients receiving concurrent chemotherapy, DMARDS were still not associated with a higher rate.

**Table 3.** Results of Univariate Analysis of Factors Associated With Acute or Late Toxicities.

	Acute Tox, Grade≥2		Acute Tox,Grade≥3		Late Tox,Grade≥2		Late Tox,Grade≥3	
Variable	N (%)	*P*	N (%)	*P*	N(%)	*P*	N(%)	***p***
Age at the time of RT, years								
<59	8 (73)	0.67	5 (46)	0.40	3 (27)	0.64	2 (18)	0.59
≥59	7 (58)		3 (25)		2 (17)		1 (8)	
Age at CVD diagnosis, years								
<50	7 (70)	1.00	4 (40)	0.69	3 (30)	0.62	1 (10)	1.00
≥50	8 (62)		4 (31)		2 (15)		2 (15)	
Sex								
Female	10 (67)	1.00	7 (47)	0.18	2 (13)	0.30	1 (7)	0.27
Male	5 (63)		1 (13)		3 (38)		2 (25)	
Race								
White	8 (62)	1.00	4 (31)	0.69	3 (23)	1.00	3 (23)	0.23
Non-white	7 (70)		4 (40)		2 (20)		0 (0)	
Smoking status								
Never	8 (67)	1.00	4 (33)	1.00	3 (25)	1.00	2 (17)	1.00
Former	7 (64)		4 (36)		2 (18)		1 (9)	
CVD type risk category								
High (SLE/DM)	6 (75)	0.66	2 (25)	0.66	2 (25)	1.00	2 (25)	0.27
Low (all others)	9 (60)		6 (40)		3 (20)		1 (7)	
CVD symptom burden at the time of RT								
None	9 (53)	0.06	5 (29)	0.62	3 (18)	1.00	2 (12)	1.00
Mild/moderate/high	6 (100)		3 (50)		2 (33)		1 (17)	
Taking DMARDs at the time of RT								
Yes	9 (60)	0.66	5 (33)	1.00	4 (27)	0.62	2 (13)	1.00
No	6 (75)		3 (38)		1 (13)		1 (13)	
Taking multiple CVD meds at the time of RT								
Yes	5 (50)	0.22	2 (20)	0.38	4 (40)	0.13	2 (20)	0.56
No	10 (77)		6 (46)		1 (8)		1 (8)	
RT setting								
Primary	8 (89)	0.09	4 (44)	0.66	3 (33)	0.34	1 (11)	1.00
Adjuvant	7 (50)		4 (29)		2 (14)		2 (14)	
Elective Nodal RT								
Yes	13 (72)	0.30	6 (33)	1.00	4 (22)	1.00	2 (11)	0.54
No	2 (40)		2 (40)		1 (20)		1 (20)	
BED-3, Gy								
<114.4					1 (10)	0.34	1 (10)	1.00
≥114.4					4 (31)		2 (15)	
BED-10, Gy								
<80.5	3 (30)	0.006	3 (30)	1.00				
≥80.5	12 (92)		5 (39)					
Planning target volume, cc (Missing N = 5)								
<65.0	5 (56)	1.00	2 (22)	1.00	3 (33)	0.58	1 (11)	1.00
≥65.0	5 (56)		2( 22)		1 (11)		1 (11)	
Concurrent chemotherapy								
Yes	9 (100)	0.007	5 (56)	0.18	3 (33)	0.34	2 (22)	0.54
No	6 (43)		3 (21)		2 (14)		1 (7)	

Total N = 23 patients.

Abbreviations: Tox, toxicities; RT, radiotherapy; CVD, collagen vascular disease; H&N, head and neck; DMARD, disease-modifying antirheumatic drug; BED, biologically-effective dose; BED-3, BED with ***α / β*** ratio of 3; BED-10, BED with ***α / β*** ratio of 10.

No variables were associated with a statistically-significant increase in the rate of late grade 2 or 3 toxicities. Factors associated with numerically, but not statistically, significant higher rate of late grade ≥ 2 toxicities were as follows: male vs. female sex (38% vs 13%, *P* = .30) and being on multiple medications for CVD management at the time of RT (36% vs 8%, *P* = .16). No variables were associated with a statistically-significant increase in rate of late grade 3 toxicities. Factors associated with numerically, but not statistically, significant higher rate of late grade 3 toxicities were as follows: male vs. female sex (25% vs 7%, *P* = .27) and high-risk CVDs (SLE/DM) versus low-risk CVDs (25% vs 7%, *P* = .27). The use of DMARDs (yes vs no) during HNC RT was not associated with a higher rate of late grade ≥ 2 toxicities (27% vs 13%, *P* = .62) or late grade ≥ 3 toxicities (13% vs 13%, *P* = 1.00; Figure 2). In patients who had received concurrent chemotherapy, the use of DMARDS was likewise not a significant factor.

## Discussion

In this study of patients with head and neck cancer and comorbid CVD treated between 2005 and 2022, definitive or postoperative IMRT was associated with approximately 35% acute and 15% late grade ≥ 3 toxicity rates. The use of concurrent chemotherapy and a higher BED was the only clinical or treatment factor statistically associated with a higher rate of acute grade ≥ 2 toxicities. There were no factors statistically associated with increased acute grade ≥ 3 toxicities or late grade ≥ 2 or ≥ 3 toxicities. Specifically, the use of concurrent DMARDs during HNC IMRT was not associated with a higher rate of acute or late toxicities.

RT plays an important role in the treatment of patients with head and neck cancer, but due to the close vicinity of the treatment target volumes to normal structures in the head and neck, treatment is often accompanied with significant toxicity. Very few studies evaluating patients with CVD receiving RT have selected for radiation site or cancer subtype. In 1989, Teo et al published outcomes for 10 patients with DM and nasopharyngeal carcinoma (NPC) treated with RT, reporting that all patients experienced indurated subcutaneous fibrosis and complete xerostomia at variable intervals after treatment, and 2 out of the 10 patients experienced radiation skin necrosis.^10^ More recently, Huang et al described a matched-pair analysis of 172 patients with NPC with or without DM treated with RT and found that patients with DM experienced a significantly-higher rate of acute toxicities, but that there were no differences in late toxicities.^11^ In a meta-analysis of 10 case-control studies evaluating patients with CVDs receiving RT, Shaikh et al found that patients with CVDs who received RT to the head and neck had a significantly-higher rate of acute grade 2-3 toxicities (OR 3.10, 95% CI 1.81-5.33; *P* < .0001) than controls, and a higher rate of late grade 2-3 toxicities that was not statistically significant (OR 1.59, 95% CI 0.88-2.90; *P* = .13).^6^ Of note, the vast majority of patients in these studies were treated prior to the introduction of current RT and imaging techniques, such as image-guided RT or IMRT. IMRT has now been demonstrated in prospective studies to provide good long-term quality of life outcomes for patients with head and neck cancer.^13-17^ In our patient cohort, which includes patients with disease involving various head and neck subsites, we found that the acute grade ≥ 3 toxicity rate was 35% and the late grade ≥ 3 toxicity rate was 13% for patients with CVD receiving IMRT to the head and neck with or without concurrent chemotherapy, indicating that severe toxicity rates for patients with CVD and head and neck cancer may be acceptable in the era of IMRT.

In the present study, we found that a higher BED and concurrent chemotherapy administration were statistically associated with a higher rate of acute grade ≥ 2 toxicities, factors that have historically been associated with increased acute toxicities in patients with head and neck cancer treated with RT.^18-22^ Although no factors were statistically associated with a higher rate of late toxicities, this could be due to the low event rate, with only 3 patients experiencing late grade 3 toxicities. We did find that a higher proportion of patients with SLE or DM had late grade 3 toxicities compared with patients who had other CVDs. This indicates that certain CVD subtypes may be associated with an increased risk for late toxicities from radiation, which has been demonstrated in meta-analyses investigating patients with CVDs treated with RT.^6^ However, this result did not reach statistical significance and would need to be confirmed with a larger patient cohort.

Since symptoms due to CVD flares can be additive to the acute toxicities from radiation treatment, it is important for radiation oncologists to work closely with patients’ rheumatologists to manage medications thoughtfully for patients with CVDs during their HNC RT course. DMARDs are commonly part of first-line therapy for patients with rheumatic arthritis and are also often used for patients with other CVDs in order to suppress the immune and/or inflammatory response. There is an overall lack in consensus across the few published guidelines regarding the use of DMARDs in patients with cancer, and the guidelines that do exist primarily advise on the use of DMARDs during systemic therapy for cancer.^23-25^ No guidelines exist regarding DMARD use during RT, and very few studies even report on the use of DMARD use during RT. Lin et al conducted a retrospective matched-control analysis including 73 patients with CVD treated with RT between 1985 and 2005, with each patient matched to 1-3 patients without CVD, and reported that the use of corticosteroids, nonsteroidal anti-inflammatory drugs (NSAIDs), statins, calcium channel blockers, and antimalarial antirheumatic drugs was not associated with increased risk of any acute or late toxicities for the patients with CVDs.^9^ In the present study of patients treated between 2005 and 2022, we provide information regarding conventional and biological DMARDs that are now more commonly used in patients with CVDs. Patients using these DMARDs at the time of IMRT were not at significantly-higher risk of acute or late toxicities. Although this will need to be confirmed with larger patient cohorts, these data indicate that the concurrent use of DMARDs may be safe with RT in patients with head and neck cancers treated with or without concurrent chemotherapy.

This study has multiple limitations. One limitation is the retrospective nature of this study with the associated risk for selection bias. For example, almost all patients had mild or no symptom burden from their CVD at the time of IMRT, and only 1 patient had a moderate-to-high CVD symptom burden at the time of IMRT, indicating that the overall CVDs in a patient cohort were relatively well-controlled at the time of radiation treatment. Furthermore, relying on rheumatologist-defined symptom burden, without standardized or disease-specific tools, may represent a potential confounder. The small sample size limited the statistical power of this study to assess for factors associated with severe treatment-related toxicities. Finally, although the patient cohort was limited to those who were treated to the head and neck with curative-intent and the treatment modality was limited to IMRT, the patient population was still heterogeneous in multiple ways, including the head and neck subsite-treated, disease histology, CVD subtype, and type of DMARDs used. Ultimately, the decision of whether to continue DMARDs during RT should be made on an individual case-by-case basis, taking into consideration the specific clinical situation.

## Conclusion

In this small cohort of patients with head and neck cancer and comorbid collagen vascular disease, definitive or postoperative IMRT was associated with approximately 35% acute and 15% late severe grade ≥ 3 toxicity rates. The use of concurrent conventional or biological DMARDs during IMRT was not associated with a higher rate of acute or late toxicities. Larger, prospective studies are necessary to validate the safety of concurrent DMARD use in patients with CVD receiving IMRT for head and neck cancer.
